# Thyroid function in children with Prader-Willi syndrome in Southern China: a single-center retrospective case series

**DOI:** 10.1186/s12887-022-03275-5

**Published:** 2022-04-29

**Authors:** Xinjiang Huang, Xi Yin, Dongyan Wu, Yanna Cai, Xiuzhen Li, Wen Zhang, Chunhua Zeng, Xiaojian Mao, Li Liu

**Affiliations:** 1 Jinan University, Guangzhou, 510632 China; 2 Department of Genetics and Endocrinology, Guangzhou Women and Children’s Medical Center, National Children’s Medical Center for South Central Region, Guangzhou Medical University, 9 Jinsui Road Tianhe District, Guangzhou, 510623 China; 3 Department of Genetics and Endocrinology Laboratory, Guangzhou Women and Children’s Medical Center, National Children’s Medical Center for South Central Region, Guangzhou Medical University, Guangzhou, 510623 China

**Keywords:** Prader-Willi syndrome, Thyroid function, Hypothyroidism, Recombinant human growth hormone

## Abstract

**Background:**

To investigate hypothalamic-pituitary-thyroid function in children of different ages, nutritional phases, and genotypes that were diagnosed with Prader-Willi syndrome (PWS), as well as the effects of recombinant human growth hormone (rhGH) treatment on thyroid hormones in PWS patients.

**Methods:**

One hundred and thirty PWS patients (87 boys and 43 girls) aged from newborn to 15 years (y) (median 1.25 y, mean, SD: 2.95 ± 3.45 y), were surveyed in this study. Serum thyroid hormone levels were examined at least once per3-6 months during the 2 years follow-up study. Central hypothyroidism (C-HT) was identified as low/normal thyroid-stimulating hormone (TSH) and low free thyroxine 4 (FT4).

**Results:**

All study participants had normal neonatal TSH screening test results. The prevalence of C-HT is 36.2% (47/130). No C-HT cases were diagnosed in PWS either below 1 month (m) or above 12 y. The prevalence of C-TH would be increased with age before 3 y until reaching the peak, followed by a gradual decline over the years. The prevalence of C-HT varies significantly at different ages (Pearson's χ2 = 19.915; *p* < 0.01). However, there is no correlation between the C-HT prevalence and nutritional phases (Pearson's χ2 = 4.992; *p* = 0.288), genotypes (Pearson's χ2 = 0.292; *p* = 0.864), or rhGH therapy (Pearson's χ2 = 1.799; *p* = 0.180).

**Conclusions:**

This study suggests the prevalence of C-TH was increased with the age before 3 y, and reached the peak in the 1 to 3 y group, then gradually declined over the years. There is no correlation between C-HT prevalence and nutritional phases, genotypes, or rhGH treatment.

## Introduction

Prader-Willi syndrome (PWS; OMIM #176,270) is a human imprinting disorder caused by the inactivation of expression of paternally transcribed genes in a highly imprinted region of chromosome 15q11-q13. PWS is caused by paternal deletion (65–70%), maternal uniparental disomy (UPD) (20–30%) and imprinting center (IC) defect (1–3%) [1–3]. Patients with paternal deletion are more related to developing feeding difficulties, sleep disturbances, hypopigmentation, higher body mass index (BMI), and speech and language deficits [4, 5]. Several other features are more common in UPD individuals, such as post-term delivery, higher verbal intelligence quotient (IQ), psychosis, and autism spectrum disorder [6]. On the other hand, UPD patients are less likely to have the typical PWS facial appearance and hypopigmentation [3]. And, it is still unclear whether hypothyroidism has a genotype-phenotype-specific correlation which is urgently needed to be studied.

PWS is characterized by the dysregulation of the hypothalamic-pituitary axis, such as hypotonia and feeding difficulties in the neonatal or even infant periods, presented hyperphagia and obesity starting in childhood; usually followed by short stature, intellectual disability, and behavioral abnormalities [3, 7–9]. So, secondary hypothyroidism which originates from abnormalities of the hypothalamic-pituitary axis, namely central hypothyroidism (C-HT), maybe more common in children with PWS. Current reports of the prevalence of hypothyroidism in children with PWS are from 2% to 72.2% worldwide, but these reports are highly inconsistent [10–15].

The clinical presentations of PWS are always age-specific. Partial hypogonadotrophic hypogonadism, one kind of hypothalamic-pituitary axis dysfunction, presents in some elder children and adults with PWS but is not seen in infancy [16]. Until puberty, luteinizing hormone (LH) levels increase but only reach the low regions of the normal range while follicle-stimulating hormone (FSH) levels are normal to high which could be interpreted as partial hypogonadotrophic hypogonadism [17, 18]. Conversely, inhibin B and Müllerian hormone levels decline to low and even undetectable levels in adulthood. This pattern is indicative of the etiology of hypogonadism, while variable, it is often due to primary testicular dysfunction [19, 20]. On the contrary, hypotonia and feeding difficulties are very common in infant PWS while symptoms will be relieved with age which has been classically described as two nutritional phases [3]. However, the feeding behaviors of PWS patients may be more gradual and complex than the traditional two phases. It is well-established by now that nutritional phases can be identified a total of seven different nutritional phases, with five main phases and sub-phases in phases 1 and 2 [21]. Besides, early rhGH therapy can increase muscle strength in the hypotonic stage, as well as improve energy expenditure and body composition [22]. While one study reported that a significant reduction of FT4 to low-normal levels was observed during rhGH treatment of PWS patients [23].

C-HT may be more common in PWS patients, yet if the prevalence is different with ages, genotypes, nutritional phases, or related to rhGH therapy remains unclear. Therefore, the goal of this study is to investigate the hypothalamic-pituitary-thyroid function in PWS children of different ages, genotypes, and nutritional phases as well as the effects of rhGH treatment on their thyroid hormone levels.

## Subjects and methods

One hundred and thirty patients (87 boys and 43 girls) with PWS, the age when PWS was diagnosed ranging from newborn to 15 y (median 1.25 y, mean, SD: 2.95 ± 3.45 y), and the age when central hypothyroidism was diagnosed ranging from 1 m to 9 y (median 1.5 y, mean, SD: 2.48 ± 2.42 y), referred to Guangzhou Women and Children's Medical Center from January 2016 to January 2020, were contained in this retrospective study. PWS diagnosis was based on molecular analyses with the clinical manifestation of hypotonia and feeding difficulties in the neonatal or even infant periods, hyperphagia, and obesity starting in childhood; usually followed by light skin color, small hands and feet, hypogonadism, short stature, intellectual disability, and behavioral abnormalities. Molecular analyses were based on methylation analysis of 15q11-q13, including methylation special polymerase chain reaction (MS-PCR) and methylation-sensitive multiplex ligation-dependent probe amplification (MS-MLPA) (Kit available from MRC Holland http://www.mrc-holland.com). Aetiology of PWS was established by MS-MLPA, fluorescence in situ hybridization (FISH), and chromosome microarray analysis (CMA) (CytoScan 750 K array, Affymetrix Inc., Santa Clara, California). The genotypes were classified as paternal 15q11-q13 deletion, UPD, IC defect, Robertsonian translocation (RT), and unclassified.

Subjects were classified according to chronological age, nutritional phases [21], genotypes, and whether or not they were receiving rhGH treatment. Thyroid-stimulating hormone (TSH), free thyroxine 4 (FT4), and free triiodothyronine 3 (FT3) were measured in our institution by a chemiluminescence immunoassay (ADVIA Centaur XP Immunoassay Systems, Siemens, Erlangen, Germany). The thyroid hormones were assessed at least once per 3–6 months during the 2 years follow-up in all patients. C-HT was identified as low/normal TSH and low FT4. L-thyroxine (Euthyrox, 1-2mcg/kg/day) replacement was initiated once C-HT was identified. The adrenal function, morning cortisol and adrenocorticotropic hormone, were always measured together with thyroid function. And, the C-HT patients were confirmed to have normal adrenal function before replacement therapy. When the weight was less than 12.5 kg (kg), we prescribed L-thyroxine at a dose of approximately 2 mcg per kg body weight; when the weight was more than 12.5 kg, we prescribed L-thyroxine at a dose of approximately 1 mcg per kg body weight. The dosage was adjusted appropriately in conjunction with the convenience of dispensing, with a minimum dose of 12.5 mcg and a maximum of 50 mcg (1 tablet). Some individuals require continuous replacement while others do not. If the levels of FT4 and TSH could be maintained in the reference range, the treatment continued. If that is out of the normal levels, accompanied by increased heart rate, sweating, dysphoria, and other thyrotoxicosis symptoms, the ones were able to stop treatment. Details of clinical data were obtained from patient medical records.

To establish reference thyroid hormone serum values, the laboratory data of the Genetics and Endocrinology laboratory of Guangzhou Women and Children's Medical Center collected between 2010 and 2013 were used. Serum samples of 1138 healthy children and adolescents, from newborn to the age of 18 y, were used to establish the reference range. All participants were informed consent obtained from their parents. Participants were divided into the following age groups: < 1 m, *n* = 195; 1 m-0.99y, *n* = 196; 1y-2.99y, *n* = 110; 3y-5.99y, *n* = 121; 6y-8.99y, *n* = 64; 9y-11.99y, *n* = 64; 12-18y, *n* = 388. Reference values are shown in Table 1. The 2.5th and the 97.5th percentiles were used as the reference interval for FT3, FT4, and TSH.

**Table 1:** Reference of serum thyroid hormone levels in the healthy population of Southern China

	**FT4(pmol/L)**	**TSH(mIU/L)**
< 1 m (*n* = 195)	12.00–29.34	0.47–10.00
1 m-0.99y (*n* = 196)	13.17–22.33	0.36–7.63
1y-2.99y (*n* = 110)	14.45–22.74	0.38–7.31
3y-5.99y (*n* = 121)	15.49–22.38	0.65–7.18
6y-8.99y (*n* = 64)	14.42–22.22	0.79–6.06
9y-11.99y (*n* = 64)	13.44–23.61	0.91–4.63
≥12y (*n* = 388)	11.5–22.7	0.51–4.94

*m* months, *y* year, *n* number

All study participants had normal neonatal TSH screening test results.

Frequency data were analyzed with detailed number and percent, continuous data were analyzed with the mean ± standard deviation scores (minimum–maximum value) with or without reference range (Ref.) and categorical data were analyzed using a chi-square test with SPSS 22.0 (SPSS Inc., Chicago, Illinois) software. For each test, statistical significance was considered for *p* < 0.05.

## Results

Overall, one hundred and thirty PWS patients were included in this study. Serum thyroid hormone levels of PWS in all groups are shown in Table 2. Forty-seven of 130 patients with PWS (47/130, 36.2%) were identified as C-HT according to low/normal TSH and low FT4. Serum thyroid hormone levels of C-HT patients in different groups are shown in Table 3. L-thyroxine (Euthyrox, 1-2mcg/kg/day) replacement was initiated as soon as C-HT was identified. All patients were followed up for at least two years. Twenty-three of 47 (23/47, 48.9%) C-TH patients needed to continue L-thyroxine treatment (treatment time; mean, SD: 28.87 ± 13.87 months) to maintain normal thyroid hormone levels while the other 20 patients (20/47, 42.6%) could stop L-thyroxine replacement after several months (treatment time; mean, SD: 9.05 ± 8.85 months). Four cases were untreated because they were lost to follow-up after diagnosis.

**Table 2:** Thyroid hormone levels of PWS in the different groups

	**FT4(pmol/L)**	**TSH(mIU/L)**	**C-HT(column**^**a**^**)**	**C-HT(row**^**b**^**)**
**Age**				
< 0.08y (*n* = 11)	18.34 ± 3.30 (12.54–23.81) Ref.(12.00–29.34)	3.19 ± 1.46 (1.36–6.42) Ref.(0.47–10.00)	0	0
0.08y-0.99y (*n* = 46)	14.17 ± 2.75 (8.58–19.14) Ref.(13.17–22.33)	3.00 ± 2.09 (0.02–9.44) Ref.(0.36–7.63)	18(38.3%)	18(39.1%)
1y-2.99y (*n* = 23)	15.38 ± 3.47 (9.19–23.01) Ref.(14.45–22.74)	2.77 ± 1.85 (0.41–8.39) Ref.(0.38–7.31)	12(25.5%)	12(52.2%)
3y-5.99y (*n* = 26)	16.71 ± 2.66 (13.10–21.18) Ref.(15.49–22.38)	2.61 ± 0.83 (1.23–4.22) Ref.(0.65–7.18)	12(25.5%)	12(46.2%)
6y-8.99y (*n* = 12)	16.52 ± 3.24 (12.06–21.75) Ref.(14.42–22.22)	2.47 ± 1.03 (0.96–4.03) Ref.(0.79–6.06)	4(8.5%)	4(33.3%)
9y-11.99y (*n* = 8)	17.40 ± 4.81 (11.28–27.61) Ref.(13.44–23.61)	2.44 ± 1.25 (0.99–4.54) Ref.(0.91–4.63)	1(2.1%)	1(12.5%)
> 12y (*n* = 4)	17.85 ± 4.50 (14.43–23.96) Ref.(11.5–22.7)	1.87 ± 0.64 (1.30–2.68) Ref.(0.51–4.94)	0	0
**Nutritional Phases Groups**				
Phase 1a (*n* = 49)	15.11 ± 3.34 (8.58–23.81)	3.01 ± 1.70 (0.23–7.38)	15(31.9%)	15(30.6%)
Phase 1b (*n* = 25)	14.89 ± 3.58 (9.19–23.01)	3.09 ± 2.50 (0.02–9.44)	12(25.5%)	12(48.0%)
Phase 2a (*n* = 23)	16.67 ± 2.38 (13.56–19.89)	2.41 ± 0.70(1.23–4.22)	10(21.3%)	10(43.5%)
Phase 2b (*n* = 17)	16.51 ± 2.96 (12.94–21.57)	2.63 ± 0.97 (1.24–4.17)	7(14.9%)	7(41.2%)
Phase 3 (*n* = 16)	17.14 ± 4.35 (11.28–27.61)	2.31 ± 1.12 (0.96–4.54)	3(6.4%)	3(18.8%)
**Genotype**				
Deletion (*n* = 84)	15.35 ± 3.15 (8.58–23.96)	2.80 ± 1.62(0.02–8.39)	31(66.0%)	31(36.9%)
UPD (*n* = 33)	16.29 ± 3.75 (11.28–27.61)	2.71 ± 1.35 (0.76–6.99)	13(27.7%)	13(39.4%)
Unclassified (*n* = 10)	16.59 ± 3.67 (11.84–23.01)	2.16 ± 1.49 (0.41–5.84)	3(6.4%)	3(30.0%)
IC defect (*n* = 2)	20.93 ± 3.42 (18.51–23.35)	2.89 ± 0.30 (2.68–3.10)	0	0
RT (*n* = 1)	15.74	9.44	0	0
**rhGH therapy**				
Yes (*n* = 73)	14.73 ± 2.80 (8.58–23.01)	2.79 ± 1.72 (0.02–8.39)	35(74.5%)	35(47.9%)^**c**^
No (*n* = 57)	17.12 ± 3.64 (9.12–27.61)	2.77 ± 1.54 (0.23–9.44)	12(25.5%)	12(22.6%)
**Total** (*n* = 130)	15.77 ± 3.40 (8.58–27.61)	2.78 ± 1.64 (0.02–9.44)	47(100%)	47(36.2%)

Data were recorded as mean ± SD (minimum–maximum) with or withoutreference range. Data of C-HT in the subgroup are presented as a number of patients and percentages according to the patients' group

*C-HT* Central hypothyroidism, *UPD* Uniparental disomy, *IC* Imprinting center, *RT* Robesonian translocation, *rhGH* Recombinant human growth hormone, *m* months, *y* year, *n* number, *Ref*. Reference range

^a^Percent of patients according to total C-HT (column)

^b^Percent of patients according to age group (row)

^c^In rhGH therapy group, 35 patients (35/73, 47.9%) were identified as C-HT, while 26 of them (26/73, 35.6%) were identified before rhGH treatment, 9 (9/73, 12.3%) were identified during rhGH therapy

**Table 3:** Thyroid hormone levels of the PWS patients with C-HT in the different groups

	**FT4(pmol/L)**	**TSH(mIU/L)**
**Age Groups**		
1 m-0.99y (*n* = 18)	11.48 ± 1.37 (8.58–13.11) Ref. (13.17–22.33)	2.48 ± 1.68 (0.02–7.38) Ref. (0.36–7.63)
1y-2.99y (*n* = 12)	12.88 ± 1.77 (9.19–14.43) Ref. (14.45–22.74)	2.55 ± 1.48 (0.41–4.86) Ref. (0.38–7.31)
3y-5.99y (*n* = 12)	14.18 ± 0.75 (13.10–15.39) Ref. (15.49–22.38)	2.78 ± 0.91 (1.28–4.22) Ref. (0.65–7.18)
6y-8.99y (*n* = 4)	13.02 ± 0.75 (12.06–13.88) Ref. (14.42–22.22)	2.05 ± 0.90 (0.96–3.09) Ref. (0.79–6.06)
9y-11.99y (*n* = 1)	11.28 Ref. (11.5–22.7)	1.53 Ref. (0.51–4.94)
**Nutritional Phases Groups**		
Phase 1a (*n* = 15)	11.59 ± 1.38 (8.58–13.11)	2.68 ± 1.70 (0.54–7.38)
Phase 1b (*n* = 12)	12.13 ± 1.89 (9.19–14.43)	2.43 ± 1.64 (0.02–4.86)
Phase 2a (*n* = 10)	14.26 ± 0.60 (13.56–15.39)	2.56 ± 0.84 (1.41–4.22)
Phase 2b (*n* = 7)	13.66 ± 0.79 (12.94–15.12)	2.54 ± 0.93 (1.28–4.13)
Phase 3 (*n* = 3)	12.41 ± 1.33 (11.28–13.88)	1.86 ± 1.10 (0.96–3.09)
**Genotype**		
Deletion (*n* = 31)	12.31 ± 1.82 (8.58–15.12)	2.51 ± 1.50 (0.02–7.38)
UPD (*n* = 13)	13.50 ± 1.14 (11.28–15.39)	2.81 ± 1.04 (1.28–4.86)
Unclassified (*n* = 3)	12.61 ± 0.95 (11.84–13.67)	1.37 ± 0.34 (1.01–1.69)
**rhGH therapy**		
Yes (*n* = 35)	12.66 ± 1.56 (8.58–14.88)	2.55 ± 1.50 (0.02–7.38)
during rhGH therapy (*n* = 9)	13.31 ± 1.14 (11.28–14.84)	2.05 ± 0.88 (0.54–3.30)
No (*n* = 12)	12.63 ± 2.07 (9.12–15.39)	2.42 ± 0.93 (0.77–4.22)
**Total C-HT(*n* = 47)**	12.66 ± 1.68 (8.58–15.39)	2.51 ± 1.37 (0.02–7.38)

Data were recorded as mean ± SD (minimum–maximum) with or without reference range

*C-HT* Central hypothyroidism, *UPD* Uniparental disomy, *IC* Imprinting center, *RT* Robesonian translocation, *rhGH* Recombinant human growth hormone, *m* months, *y* year, *n* number, *Ref.* Reference range

**Fig. 1:**
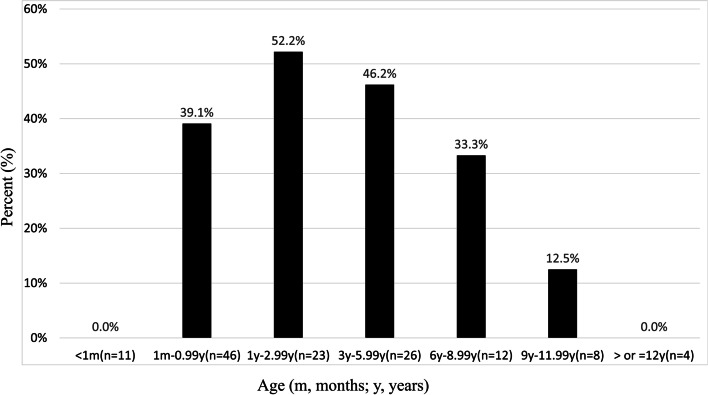
The PWS patients with C-HT in different age groups. Subjects were divided into seven groups according to their chronological ages. The prevalence of C-TH is increased with the age group before 3 y, reached the peak in the 1 to 3 y group, and gradually declined over the years. The prevalence of C-HT varies significantly between different age groups (Pearson's χ2 = 19.915; *p* < 0.01). m, months; y, years; C-HT, central hypothyroidism

We have compared the prevalence of C-TH in different age groups (Table 2). The prevalence of C-HT in aged-subgroups was significantly different between 1 m to 0.99 y (39.1%; 18/46), 1 y to 2.99 y (52.2%; 12/23), 3 y to 5.99 y (46.2%; 12/26), 6 y to 8.99 y (33.3%; 4/12), 9 y to 11.99 y (12.5%; 1/8) (Pearson's χ2 = 19.915; *p* < 0.01). However, no C-HT cases were identified either below 1 m or above 12 y. Also, it should be noted that a normal TSH screening (without T4 measurements) does not rule out C-HT and this may be why no infants below the age of one month were diagnosed as having C-HT. The prevalence of C-TH increased with age before 3 y and reached the peak in the 1 to 3 y group, then gradually declined over the subsequent age groups (Fig. 1).

**Fig. 2:**
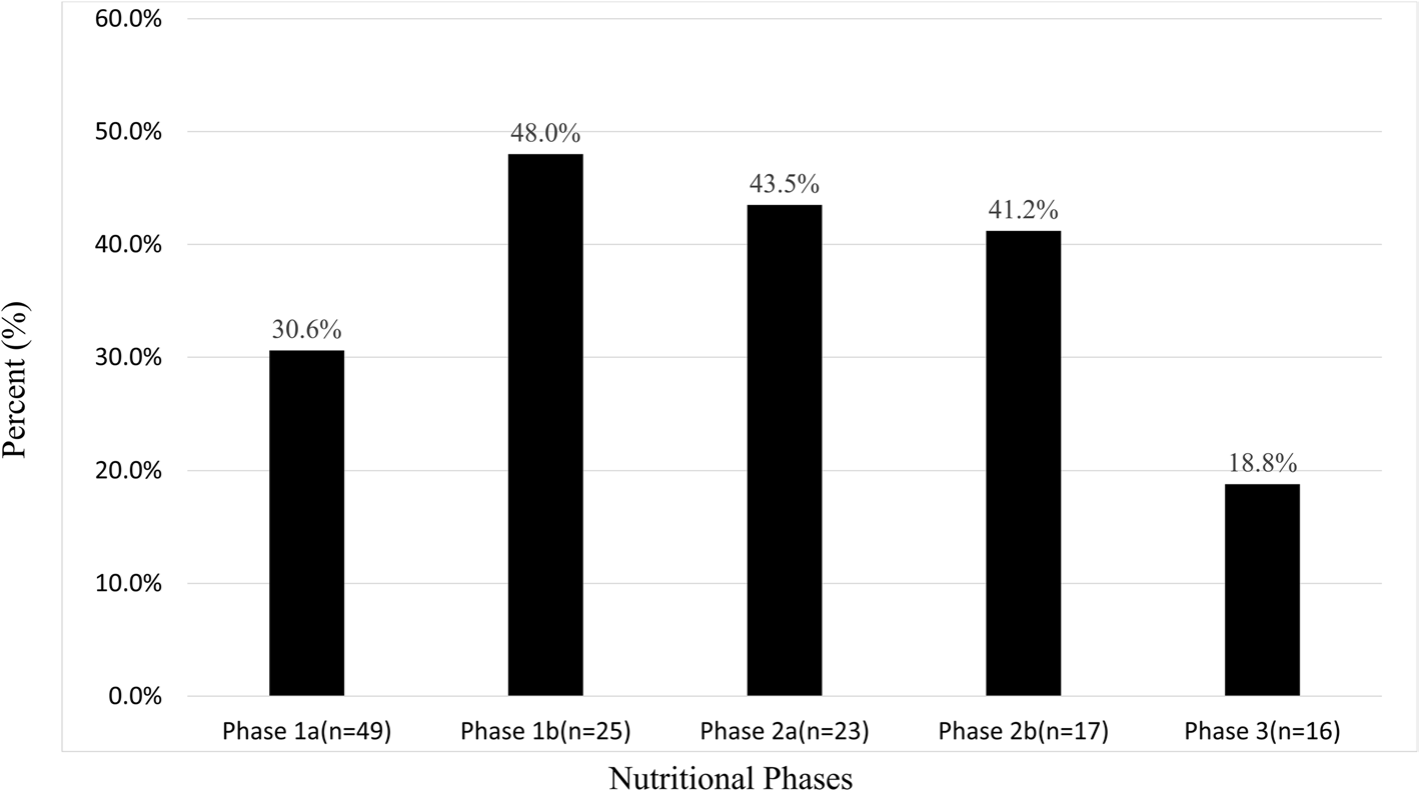
The PWS patients with C-HT in different nutritional phase groups. Subjects were divided into seven groups according to their nutritional phase groups. The prevalence of C-HT was no significantly different between different nutritional phase groups (Pearson's χ2 = 4.992; *p* = 0.288). C-HT, central hypothyroidism

We compared the prevalence of C-TH in different nutritional phase groups (Table 2 and Fig. 2) according to the prefer report [21]. The prevalence of C-HT in nutritional phases-subgroups was no significantly different between phase 1a(30.6%; 15/49), phase 1b (48.0%; 12/25), phase 2a (43.5%; 10/23), phase 2b (41.2%; 7/17), phase 3 (18.8%; 3/16) (Pearson's χ2 = 4.992; *p* = 0.288).

Eighty-four (84/130, 64.6%) patients had a paternal 15q11-q13 deletion, 33 (33/130, 25.4%) were maternal UPD, 2 (2/130, 1.5%) were IC defect, 1 (1/130, 0.8%) was RT and 10 (10/130, 7.7%) were unclassified (confirmed diagnosis by methylation analysis without further classification). The prevalence of C-HT in genotype-subgroups was no significantly different between paternal deletion (36.9%; 31/84), UPD (39.4%; 13/33) and unclassified groups (30%; 3/10) (Pearson's χ2 = 0.292; *p* = 0.864). No C-HT cases were identified in either the IC defect or RT groups.

The correlation of rhGH therapy and thyroid hormone levels in PWS patients were then evaluated. Among the 130 patients, 73 patients were given rhGH therapy. The initial dosage of rhGH was 0.5 mg/m^2^/day and it was adjusted according to the levels of insulin-like growth factor 1 (IGF-1), up to 1 mg/m^2^/day. Twenty-six C-HT (26/73, 35.6%) were identified as C-HT in the rhGH therapy group before rhGH treatment. Nine patients (9/73, 12.3%) were identified as C-HT in the rhGH therapy group during rhGH treatment(treatment time; mean, SD: 3.89 ± 4.04 months). Twelve of 57 were identified as C-HT in the non-rhGH therapy group (12/57, 21.1%). So, in rhGH subgroups, the prevalence of C-HT was not significantly different between the rhGH therapy and untreated groups (Pearson's χ2 = 1.799; *p* = 0.180).

## Discussion

In this study, 47 of 130 patients with PWS (36.2%) were identified as C-HT and C-HT incidence increased with the age before 3 y until reaching the peak, followed by a gradual decline over the years; in addition, the thyroid hormone levels might be normalized with the age. However, there was no correlation between the C-HT prevalence and nutritional phases, genotypes, or rhGH therapy.

There are inconsistent results in the reports regarding the prevalence of hypothyroidism in patients with PWS. Some studies report a prevalence of 2–4% [11, 14], while others report a rate as high as 20–34.69% of the patients with PWS [10, 12, 15]. Even more, one study reported a prevalence of 72.2% of central hypothyroidism in infants and toddlers aged less than 2 y [13]. These studies revealed that the prevalence of hypothyroidism in PWS patients is variable and inconclusive. In our study, the prevalence of C-HT, the most common type of hypothyroidism in PWS patients [24], presented by 36.2% which is higher than most of the previous reports [10–12, 14, 15]. Replacement treatment was initiated as soon as C-HT was identified and all patients were followed up for at least two years. In 23 of 47 C-TH patients (23/47, 48.9%) treatment need to be continued to maintain normal thyroid hormone levels while in the other 20 patients (20/47, 42.6%) L-thyroxine treatment could be withdrawn after several months (treatment time; mean, SD: 9.05 ± 8.85 months). The discrepant prevalence compared to previous reports may be associated with the different ages of patients and methods used.

Some studies evaluated thyroid function by a thyrotropin-releasing hormone (TRH) test [10, 14, 25], while others were diagnosed through serum thyroid hormones levels [11, 13, 24]. Generalized hypothalamic insufficiency is the main characteristic of PWS [9]. PWS patients always manifest with dysfunction of the hypothalamic-pituitary axis, including growth hormone deficiency, hyperphagia, abnormal thyroid function, temperature instability, sleep disorders, and partial hypogonadotrophic hypogonadism [3]. Although whole hypothalamic-pituitary axis function assessment is indispensable to identify combined pituitary hormone deficiencies, a TRH test is not essential and could not rule out central hypothyroidism, and the diagnosis should be made by regular thyroid hormone monitoring [26]. Furthermore, patients who had a positive TRH test presented with normal TSH, FT4, and FT3 levels in the subsequent two years' follow-up without treatment [25]. This suggests that regular thyroid hormone monitoring is necessary in the long-term management, but not TRH testing. In our study, the thyroid hormone levels were assessed at least once per 3–6 months during the 2 years follow-up. Patients were identified as C-HT when hormone showed low/normal TSH and low FT4. Thus, L-thyroxine replacement was prescribed when C-HT was identified.

For the age of subjects, the prevalence of hypothyroidism is higher in infant PWS patients [13, 24], than in elder children [11, 14]. Vaiani et al. reported a high prevalence of 72.2% of C-HT in infants and toddlers below 2 y, which suggested that thyroid dysfunction may be present in many young children with PWS [13]. Iughetti et al. reported that 43.5% of C-HT were present in children younger than 1 y [24]. In our study, subjects were divided into seven groups according to age. It was found that the prevalence of C-TH increased with age before 3 y, reached the peak in the 1 to 3 y group, then gradually declined over the years (Fig. 1). No C-HT cases were identified either below 1 m or above 12 y in the current study. This finding suggests that hypothyroidism may not be present at the neonatal stage but may occur later and its incidence increases with age after the neonatal period until reaching the peak at preschool age. As the incidence of thyroid axis dysfunction was varying with age in our study, which is different from previous reports, these groupings may be appropriate to demonstrate variability by age. The thyroid function of PWS patients might be normalized with age. Similar changes occur in infants who manifest hypotonia and feeding difficulties, with these symptoms improving with age [3, 7].

As mentioned in the introduction, the clinical manifestations of PWS are age-specific. For example, some symptoms may appear in infancy while they can slowly resolve with age, such as hypotonia and feeding difficulties [3]. Maybe, hormonal changes in PWS may also be age-specific. Infancy boys with PWS usually have normal mini-puberty according to the normal testosterone, LH and FSH levels [16]. Until puberty, LH levels increase but only reach the low regions of the normal range and pubertal development is incomplete or spontaneous arrest in most cases [17, 18, 27]. This study showed that the prevalence of C-HT is highly in a specific age (1 to 3 y group) then gradually declined over the years, suggesting that thyroid hormone changes in PWS may also be age-specific. Therefore, the mechanism of this phenomenon needs further study. In this study, some C-TH patients needed to continue L-thyroxine treatment while the others could stop after several months. The possible reason may be that the thyroid function of PWS patients might be normalized or fluctuated with age.

Several studies paid attention to thyroid function in patients with PWS during rhGH therapy [23, 25, 28]. Festen et al. reported that a significant reduction of FT4 to low-normal levels was observed during rhGH treatment in 75 children with PWS [23]. However, Oto et al. evaluated thyroid function in 51 patients with PWS before and during rhGH therapy and showed no changes during the 2 years after initiating rhGH therapy in most patients [25]. In our study, the prevalence of C-HT was not significantly different between rhGH therapy and non-rhGH therapy patients. It is recommended that thyroid hormone levels should be evaluated both before and during rhGH treatment [22, 29]. In addition, we have also analyzed the prevalence of C-HT between nutritional phases and genotypes and there was no significant difference.

There are several limitations of this study. First, this was a retrospective single-center study. Certainly, a longitudinal multi-center study would provide more information to explore the prevalence of C-HT in PWS patients. Second, we did not collect the body mass index (BMI) values of PWS patients. Krause et al. found that FT4 was negatively correlated with BMI-z score and fat mass [30]. The increased weight gain observed in PWS children during transition from infancy to childhood might explain the lower FT4 levels which perhaps do not truly indicate central hypothyroidism. However, nutritional phases somewhat reflect the BMI of PWS patients. We compared the prevalence of C-HT between different nutritional stages and there was no significant difference. Third, we did not analyze FT3 levels in C-HT patients. Konishi et al. reported that although FT4 levels were low in PWS infants, FT3 levels were normal and therefore treatment with thyroid hormone replacement might not be needed [31].

## Conclusions

In summary, the current study suggested that the C-TH incidence increased with age after the neonatal period until reaching the peak at preschool age; in addition, the thyroid function might be normalized with age. There was no correlation between C-HT prevalence and nutritional phases, genotypes, or rhGH treatment. Therefore, we recommend performing regular thyroid hormone monitoring during the long-term management of PWS. L-thyroxine replacement should be prescribed when C-HT has been identified. It should be confirmed that patients have normal adrenal function before replacement therapy. Further studies are needed to explore thyroid function in PWS patients.

## Data Availability

Not applicable.

## Abbreviations

C-HT: Central hypothyroidism
CMA: Chromosome microarray analysis
FISH: Fluorescence in situ hybridization
FT4: Free thyroxine 4
FT3: Free triiodothyronine 3
FSH: Follicle-stimulating hormone
IC: Imprinting center
LH: Luteinizing hormone
MS-MLPA: Methylation-sensitive multiplex ligation-dependent probe amplification
MS-PCR: Methylation-special PCR
PWS: Prader-Willi syndrome
rhGH: Recombinant human growth hormone
RT: Robertsonian translocation
TSH: Thyroid-stimulating hormone
UPD: Maternal uniparental disomy
